# Orbital Cellulitis Following Uncomplicated Glaucoma Drainage Device Surgery: Case Report and Review of Literature

**DOI:** 10.18502/jovr.v15i3.7460

**Published:** 2020-07-29

**Authors:** Cindy X. Zheng, Joshua H. Uhr, Jordan D. Deaner, John Anhalt, Michael M. Lin, Stephen J. Moster, Reza Razeghinejad

**Affiliations:** ^1^Glaucoma Research Center, Wills Eye Hospital, Philadelphia, PA, USA

**Keywords:** Ahmed Tube Shunt, Orbital Cellulitis, Glaucoma Drainage Device

## Abstract

**Purpose:**

Orbital cellulitis (OC) is a rare postoperative complication of glaucoma drainage device (GDD) implantation. To date, there have only been 10 reported cases of OC following GDD implantation.

**Case Report:**

Here, we report a case of OC in a 57-year-old man who developed pain, proptosis, and limited extraocular motility two days after uneventful Ahmed FP7 implantation in the right eye. Contrast-enhanced computed tomography of the orbits demonstrated fat stranding and a small fluid collection, consistent with OC. He had minimal improvement with intravenous antibiotics and ultimately underwent GDD explantation. A systematic review of the literature showed that the development of OC following GDD implantation can occur in the early or late postoperative period. Immediate hospitalization with intravenous administration of broad-spectrum antibiotics is recommended. Explantation of the infected GDD is often required for source control.

**Conclusion:**

OC is a rare postoperative complication of GDD implantation. Prompt evaluation and treatment are required, often combined with GDD explantation.

##  INTRODUCTION

Glaucoma drainage devices (GDDs) are surgical devices commonly implanted in eyes with refractory glaucoma. The development of orbital cellulitis (OC) following GDD implantation is rare, with only 10 reported cases in the literature.^[1,2,3,4,5,6,7,8,9]^ Here, we report a case of OC following the placement of an Ahmed FP7 (New World Medical, Rancho Cucamonga, CA) in a 57-year-old man who showed minimal improvement with intravenous (IV) antibiotics and ultimately underwent GDD explantation.

**Figure 1:**
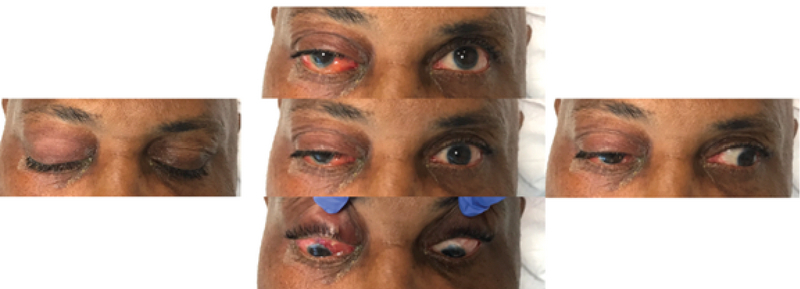
External photograph of the affected eye on the day of presentation, demonstrating periorbital edema, erythema, conjunctival injection, and chemosis.

**Figure 2:**
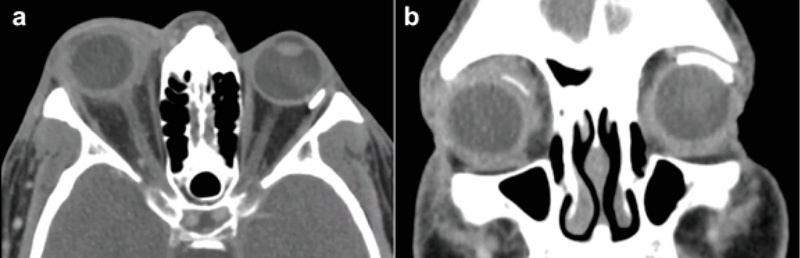
Contrast-enhanced computed tomography of the orbits showing findings consistent with orbital cellulitis, including proptosis of the right globe, thickening of the sclera and optic nerve insertion, small superior fluid collection, and mild anterior retrobulbar fat stranding (A). An Ahmed tube shunt and Baerveldt tube shunt are visualized in the right and left globes, respectively (B).

##  CASE REPORT

A 57-year-old incarcerated man with advanced primary open-angle glaucoma was referred to us because of poorly controlled intraocular pressure (IOP). He had a surgical history of bilateral trabeculectomy and implantation of a Baerveldt tube shunt implanted in the left eye approximately nine years ago. He had a medical history of gastroesophageal reflux disease and was not on any routine medications other than his glaucoma medications. On presentation, his visual acuity (VA) was 20/200 in the right eye and light perception in the left eye. His IOP was 13 mmHg in the right eye and 12 mmHg in the left eye on maximum topical therapy and oral acetazolamide. Due to difficulty tolerating acetazolamide, he agreed to proceed with Ahmed FP7 implantation in the right eye. GDD was implanted uneventfully with Tutoplast processed sclera patch graft (Katena Products Inc., Denville, NJ) in the superonasal quadrant because of conjunctival scarring from prior trabeculectomy. No intraoperative injections or mitomycin C were given. On postoperative day 1, he had a VA of 20/200 and an IOP of 10 mmHg, and the tube shunt was covered and well-positioned.

The patient presented emergently on postoperative day 4 because of two days of right eye pain, swelling, and blurry vision. He reported that he did not receive his postoperative topical ofloxacin or prednisolone acetate drops from his facility. VA was hand motion and IOP was 20 mmHg. Externally, the right orbit was tense with lid erythema and edema. His right globe was proptotic with limited extraocular motility (Figure 1). There was a small opening in the conjunctiva over the patch graft, located 4 mm posterior to the limbus. A sample of purulent drainage from this opening was swabbed and sent for microbiologic testing. The anterior chamber was deep with rare cells. There was no vitritis. Contrast-enhanced computed tomography (CT) demonstrated soft-tissue thickening, fatty infiltration, and a small fluid collection superiorly (Figure 2).

He was admitted for administration of IV vancomycin and piperacillin-tazobactam and topical fortified vancomycin and tobramycin. On hospitalization day 2, he received 8 mg of IV dexamethasone. Improvement was minimal with the administration of IV antibiotics for 24 hours; therefore, surgical explantation of the GDD was performed.

Intraoperatively, there was an area of conjunctival melt over the tube with pockets of purulent material surrounding the valve. To prevent intraocular penetration of the infected material into the anterior chamber, a purse-string suture was passed around the tube entry site in the sclera and was tied-off while a surgical assistant withdrew the tube. The plate and tube were noted to be completely free, presumably because of the surrounding scleritis. The implant was removed, and the area was copiously irrigated with vancomycin and ceftazidime solution. After conjunctival closure with 8-0 Vicryl sutures, subconjunctival injections of vancomycin and ceftazidime were administered. Considering the patient's monocular status with advanced glaucoma in the affected eye and a history of poorly controlled IOP, concomitant MicroPulse transscleral cyclophotocoagulation (Iridex Corp., Mountain View, CA) was performed for 140 sec to the inferior globe at a power of 2000mW and duty cycle of 31.3%.

Cultures showed light growth of methicillin-susceptible *Staphylococcus aureus* and *Cutibacterium acnes* (formerly *Propionibacterium acnes*). He was discharged two days after the tube shunt explantation with oral moxifloxacin 400 mg and topical fortified vancomycin and gatifloxacin.

Six months after the surgery, VA was hand motion and IOP was 12 mmHg with three topical glaucoma medications. He had complete resolution of orbital signs.

##  DISCUSSION

A systematic literature review revealed a total of 11 cases of OC following GDD surgery, including the present case (Table 1). Most patients presented within two days of symptom onset.^[1,2,3,4,5,6,7,8]^ On presentation, all patients had eyelid erythema and edema, and most patients had chemosis, proptosis or globe displacement, and limited extraocular motility.

The most common GDD associated with post-implantation OC was the Ahmed valve (n = 7),^[2,3,4,5,6,7]^ although Molteno,^[1]^ Krupin–Denver,^[2]^ and Baerveldt^[8,9]^ implants have also been associated with post-implantation OC. In seven cases, symptoms of OC started in the immediate postoperative period (≤ 3 months after the surgery).^[1,2,5,6,7,8]^ In the other four cases, OC developed after the postoperative month 3.^[2,3,4,9]^ In three of the four cases of delayed-onset OC,^[2,4,9]^ the tube was exposed, presumably serving as a conduit for bacteria to travel from the ocular surface into the orbit. In one case of delayed-onset OC, the tube was not specifically exposed; however, the patient had concurrent endophthalmitis.^[3]^ The authors theorized that organisms may have gained entry into the eye from the ocular surface and OC from drainage via the tube.^[3]^

Table 2 summarizes the management of OC. CT is the imaging modality of choice for OC and was the most common modality used.^[10]^ All patients were hospitalized and administered IV antibiotics. Although the choice of antibiotic varied, the consensus was to start with broad-spectrum coverage. In the present case, vancomycin was used owing to previous studies showing a high prevalence of methicillin-resistant *Staphylococcus aureus* isolated from ocular infections.^[11]^ The antibiotic coverage changed based on infectious disease consultation or culture sensitivities. Topical antibiotics were commonly used.^[1,2,3,4,5,7,8]^

Although not routinely administered, IV steroids were used in two cases after the administration of IV antibiotics for 24 hours, including our case.^[9]^ We administered steroids under the guidance of oculoplastic consultation to reduce orbital inflammation and to improve the ease of access during GDD explantation. Previous studies have shown that steroids can help reduce the cytokine load and improve outcomes in bacterial OC.^[12,13]^

**Table 1:** Demographics, baseline clinical characteristics, and initial presentation of orbital cellulitis after glaucoma drainage device implantation


**Case No.**	**Author**	**Age**	**Gender**	**Glaucoma diagnosis**	**GDD Type**	**Location**	**Baseline VA**	**Presenting VA**	Interval${}^{*}$	**Duration${}^{†}$**	**Chemosis**	**Proptosis**	**EOM limitation**
1	Karr^[1]^	1	M	Congenital	Molteno	ST	NR	NR	1 m	1 d	Yes	Yes	Yes
2	Chaudhry^[2]^	11	F	Congenital	Krupin–Denver	ST	CF	LP	9 d	1 d	Yes	Yes	Yes
3	Chaudhry	1	F	Congenital	Ahmed model NR	NR	NR	FF	8 m	2 d	No	No	Yes
4	Kassam^[3]^	3	M	Congenital	Ahmed FP7	NR	NR	NR	8 m	2 d	Yes	Yes	NR
5	Farid^[4]^	1	F	Congenital	Ahmed model NR	NR	NR	NR	10 m	< 1 d	Yes	No	Yes
6	Esporcatte^[5]^	1	M	Congenital	Ahmed FP7	NR	NR	NR	1 m	2 d	Yes	Yes	NR
7	Marcet^[6]^	44	M	Uveitic	Ahmed model NR	Superior	CF	CF	1 d	< 1d	Yes	Yes	Yes
8	Goldfarb^[7]^	81	F	POAG	Ahmed model NR	ST	20/200	CF	1 d	< 1d	Yes	Yes	Yes
9	Zheng	57	M	POAG	Ahmed FP7	SN	20/200	HM	4 d	2 d	Yes	Yes	Yes
10	Beck^[8]^	53	M	POAG	Baerveldt 350 mm${}^{2}$	NR	20/32	20/60	3 m	1 d	Yes	Yes	Yes
11	Lavina^[9]^	78	F	CACG	Baerveldt 350 mm${}^{2}$	NR	20/400	NLP	15 m	3 d	Yes	No	Yes
${}^{*}$Interval of time between GDD implantation and presentation of OC ${}^{†}$Duration of symptoms prior to presentation GDD, glaucoma drainage device; VA, visual acuity; EOM, extraocular movement; M, male; F, female; ST, superotemporal; SN, superonasal; NR, not reported; CF, count fingers; HM, hand motion; LP, light perception; NLP, no light perception; FF, fixes and follows; d, days; m, months												

**Table 2:** Medical and surgical management of orbital cellulitis after glaucoma drainage device implantation


**Case No.**	**IV Antibiotics**	**Topical antibiotics**	**Oral antibiotics, duration**	**Tube erosion**	**Tube explant**	**Time to explant**	**Intraoperative antibiotics**	**Blood culture**	**Endo-phtha-lmitis**	**Intravitreal antibiotics**	**Culture site**	**Culture organism**
1	Cefuroxime	Tobramycin	Y, NR	Y	Y	1 d	Irrigation with gentamicin	Neg	N	N	C, D, GDD	Group A streptococcus, staphylococcus epidermidis
2	Gentamicin, cefazolin, switched to flucloxacillin, cefotaxime	Gentamicin	Ceclor for 10 d	N	N	NA	NA	NA	N	N	NA	NA
3	Ceftriaxone, gentamicin	Vancomycin, gentamicin	Ceclor for 10 d	Y	Y	5 d	IC and SC vancomycin and amikacin	NA	N	N	AC, D	No growth
4	Vancomycin, ceftazidime	Gatifloxacin	Levofloxacin for 21 d	N	Y	1 d	SC gentamicin	Neg	Y	Vancomycin, ceftazidime	C, D, V, S	Streptococcus pneumoniae
5	Vancomycin, ceftazidime, metronidazole	Moxifloxacin	NR	Y	Y	1 d	Irrigation with gentamicin, Povidone; SC vancomycin, ceftazidime	Neg	Y	Vancomycin, ceftazidime	GDD, V	No growth
6	Vancomycin, cefepime	Gatifloxacin	Amoxicillin- clavulanic acid for 10 d	N	Y	Same day	Sub-Tenon's vancomycin, ceftazidime	Neg	Y	Vancomycin, ceftazidime	GDD, V	Staphylococcus epidermidis
7	Ampicillin-sulbactam	NR	Ciprofloxacin (duration NS)	N	N	NA	NA	NA	N	N	NA	NA
8	Vancomycin, ceftriaxone, switched to piperacillin-tazobactam	Moxifloxacin	Ciprofloxacin (duration NS)	N	Y	2 d	SC vancomycin, ceftazidime	Neg	N	N	GDD	Pseudomonas aeruginosa
9	Vancomycin, piperacillin-tazobactam	Vancomycin, tobramycin	Moxifloxacin for 7 d	Y	Y	2 d	Irrigation with vancomycin, ceftazidime; SC vancomycin, ceftazidime	NA	N	N	C, D	Staphylococcus aureus, cutibacterium acnes
10	Amoxicillin-clavulanic acid	Levofloxacin	Flucloxacillin and amoxicillin-clavulanic acid in 10 d	N	N	NA	NA	NA	N	N	C	No growth
11	Ampicillin-sulbactam	NR	NR	Y	Y	NA	NA	NA	N	N	NA	No growth
NS, not specified; NR, not reported; Y, yes; N, no; d, day; Neg, negative; GDD, glaucoma drainage device; IC, intracameral; C, conjunctiva; D, discharge; AC, anterior chamber; V, vitreous; S, sutures; NA not applicable												

**Table 3:** Outcomes and additional surgical intervention after resolution of orbital cellulitis after glaucoma drainage device implantation


**Case No.**	**Follow-up**	**Visual acuity**	**Intraocular pressure**	**Complication**	**Additional surgical intervention**
1	NR	NR	NR	None	NA
2	1 year	Hand motions	7	None	NA
3	7 years	20/60	16	None	NA
4	1 month	Locate candy bars at 8 inches	NR	Retinal detachment	2 retina surgeries
5	1 month	Fixes and follows	19	Elevated intraocular pressure	Cyclophotocoagulation
6	NR	Phthisis bulbi	NR	Phthisis bulbi	NR
7	13 months	20/200	NR	Re-exposure of GDD	Revision of GDD
8	4 months	20/100	NR	None	NA
9	6 months	Hand motions	12	None	Cyclophotocoagulation at same time as GDD explantation
10	2 months	20/32	28	Elevated intraocular pressure	Cyclophotocoagulation
11	NR	Count fingers	NR	None	NA
NR, not reported; NA, not applicable; GDD, glaucoma drainage device					

Blood cultures were negative in all tested cases.^[1,3,4,5,7]^ Presumably, the infection remained localized to the orbit. Since all cases presented within three days of symptom onset, the infection was rapidly managed with antibiotics, thus, bacteremia was less likely to occur.

The GDD was surgically explanted in 8 of the 11 cases,^[1,2,3,4,5,7,9]^ including all five cases of tube exposure^[1][2,4,9]^ and all three cases of concurrent endophthalmitis.^[3,4,5]^ Presumably, erosion allowed bacteria to seed the GDD, and the infection may be difficult to clear without explanting the infected GDD. Explantation was performed in all children aged less than three years.^[1][2][3,4,5]^ In this age group, it is difficult to perform an examination without the use of general anesthesia; therefore, it may be safer to perform explantation at the initial examination than to subject the patient to repeated episodes of anesthesia. In all cases of explantation, GDDs were most frequently explanted within one to two days of presentation, suggesting that failure to respond to antibiotics can be quickly identified. During explantation, the area surrounding the tube was irrigated with antibiotics,^[1,4]^ or subconjunctival or sub-Tenon's injection of antibiotics were performed.^[2][3,4,5][7]^ In three cases, there was a sufficient improvement with IV antibiotics alone; consequently, no surgical interventions were undertaken.^[2,6,8]^

When IV antibiotics were transitioned to oral antibiotics, fluoroquinolones were most commonly used,^[4][7][8,9]^ likely owing to their vitreous penetration and relatively broad coverage.^[14]^ The duration was most commonly 10 days, as seen in four cases.^[2,5,8]^ The duration of the therapy was likely associated with the severity of presentation and response to therapy. Table 3 details the outcomes and additional procedures that were performed.

In conclusion, OC is a rare postoperative complication of GDD implantation. Immediate hospitalization with administration of broad-spectrum IV antibiotics is recommended. Explantation of the GDD is often required for source control.
